# Crystal structure and Hirshfeld surface analysis of (*E*)-2-{[(2-iodo­phen­yl)imino]­meth­yl}-6-methyl­phenol

**DOI:** 10.1107/S2056989020011974

**Published:** 2020-09-04

**Authors:** Sevgi Kansiz, Tuggan Agar, Necmi Dege, Onur Erman Dogan, Ruby Ahmed, Eiad Saif

**Affiliations:** a Samsun University, Faculty of Engineering, Department of Fundamental Sciences, 55420, Samsun, Turkey; b Yeditepe University, Department of Chemical Engineering, 34755, İstanbul, Turkey; c Ondokuz Mayıs University, Faculty of Arts and Sciences, Department of Physics, 55139, Samsun, Turkey; d Ondokuz Mayıs University, Faculty of Arts and Sciences, Department of Chemistry, 55139, Samsun, Turkey; e Department of Applied Chemistry, ZHCET, Aligarh Muslim University, Aligarh, 202002, UP, India; f Department of Computer and Electronic Engineering Technology, Sana’a Community, College, Sana’a, Yemen

**Keywords:** crystal structure, 2-iodo­phen­yl, Schiff base, Hirshfeld surface analysis

## Abstract

The title compound, C_14_H_12_INO, was synthesized by condensation of 2-hy­droxy-3-methyl­benzaldehyde and 2-iodo­aniline, and crystallizes in the ortho­rhom­bic space group *P*2_1_2_1_2_1_. The 2-iodo­phenyl and benzene rings are twisted with respect to each other, making a dihedral angle of 31.38 (2)°. The mol­ecular structure is stabilized by an O—H⋯N hydrogen bond, forming an *S*(6) ring motif. In the crystal, mol­ecules are linked by C—H⋯π inter­actions, resulting in the formation of sheets along the *a*-axis direction. Within the sheets, very weak π–π stacking inter­actions lead to additional stabilization. The Hirshfeld surface analysis and fingerprint plots reveal that the crystal structure is dominated by H⋯H (37.1%) and C⋯H (30.1%) contacts. Hydrogen bonding and van der Waals inter­actions are the dominant inter­actions in the crystal packing. The crystal studied was refined as a two-component inversion twin.

## Abstract 2

In the crystal, mol­ecules are linked by C—H⋯π inter­actions, resulting in the formation of sheets along the *a*-axis direction. Within the sheets, very weak π–π stacking inter­actions occur. The Hirshfeld surface analysis and fingerprint plots reveal that the crystal structure is dominated by H⋯H (37.1%) and C⋯H (30.1%) contacts.

## Chemical context   

Imines derived from *o*-hy­droxy aromatic carbonyls are of inter­est because of their ability to form an asymmetric intra­molecular hydrogen bond between the oxygen atom of the hydroxyl group and the nitro­gen atom of the imine moiety (Dominiak *et al.*, 2003 ▸). This ability has a decisive impact on the biological and thermo- or photochromic properties of *o*-hy­droxy aromatic Schiff bases and makes them very useful compounds in chemistry, biochemistry, medicine, and technology (Vlad *et al.*, 2018 ▸; Bouhidel *et al.*, 2018 ▸; Faizi *et al.*, 2020*a* ▸,*b* ▸). A very important issue is determining the positions of tautomeric equilibria in these compounds and various instrumental research techniques are used to provide insight into the structure of mol­ecules of studied o-hy­droxy Schiff bases (Wojciechowski *et al.*, 2003 ▸; Faizi *et al.*, 2020*c* ▸,*d* ▸).
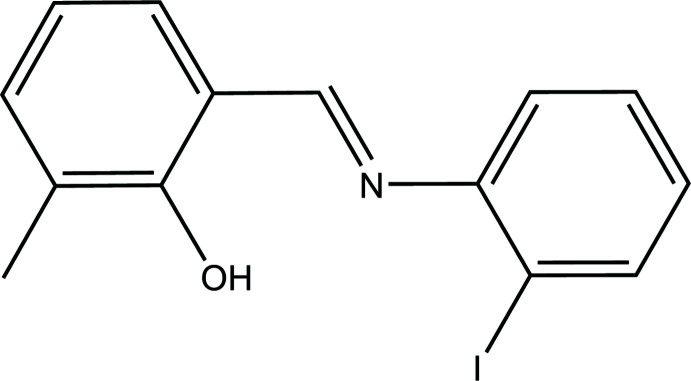


In the present study, a new Schiff base, (*E*)-2-{[(2-iodo­phen­yl)imino]­meth­yl}-6-methyl­phenol, was obtained in crystalline form from the reaction of 2-hy­droxy-3-meth­yl­benzaldehyde with 2-iodo­aniline. We report here the synthesis and the crystal and mol­ecular structures of the title compound, along with the results of a Hirshfeld surface analysis.

## Structural commentary   

**Figure 1:**
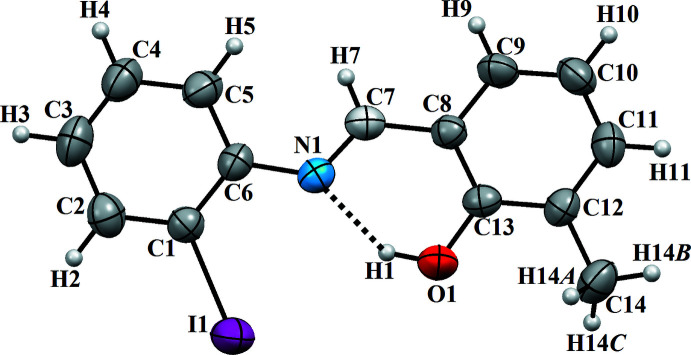
The mol­ecular structure of the title compound, with atom labelling. The intra­molecular N—H⋯O hydrogen bond (Table 1 ▸) is indicated by a dashed line. Displacement ellipsoids are drawn at the 40% probability level.

Depending on the tautomers, two types of intra­molecular hydrogen bonds are observed in Schiff bases: O—H⋯N in enol–imine and N—H⋯O in keto–amine tautomers. Most of these compounds are non-planar. The title compound, (I), is a Schiff base derivative from 2-hy­droxy-3-methyl­benzaldehyde, which crystallizes in the phenol–imine tautomeric form with an *E* configurationfor the imine functionality. The asymmetric unit of (I) contains one mol­ecule (Fig. 1 ▸). The mol­ecule is non-planar with the 2-iodo­phenyl and benzene rings twisted with respect to each other at a dihedral angle of 31.38 (2)°. The hydroxyl H atom is involved in a strong intra­molecular O—H⋯N hydrogen bond, forming an *S*(6) ring motif, which stabilizes the mol­ecular structure and induces the Schiff base atoms (N1, C7) to be coplanar with the methyl­phenol moiety. Of this planar unit (r.m.s deviation = 0.0274 Å), atoms O1 and N1 show the largest deviations from planarity in positive and negative directions [O1 = 0.035 (4) Å and N1 = −0.060 (4) Å]. The C7—N1 and C13—O1 bonds of the title compound are the most important indicators of the tautomeric type. The C13—O1 bond is of double-bond character for the keto–amine tautomer, whereas this bond displays single-bond character in the enol–imine tautomer. In addition, the C7—N1 bond is also a double bond in the enol–imine tautomer and a single bond length in the keto–amine tautomer. In the title compound, the enol–imine form is favored over the keto-amine form, as indicated by the C13—O1 [1.352 (6) Å] and C7—N1 [1.286 (8) Å] bonds, whose lengths indicate a high degree of single-bond and double-bond character, respectively. The shortest C—C distance (C3—C4) is 1.344 (11) Å in the C1–C6 ring with the weighted average ring bond distance being 1.376 (11) Å for this ring.

## Supra­molecular features   

**Figure 2:**
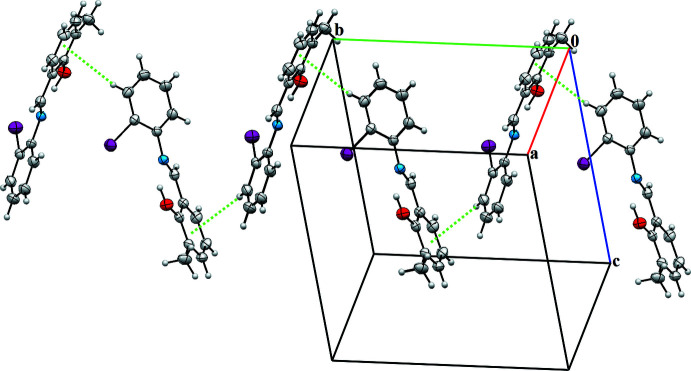
A view of the crystal packing of the title compound in a view parallel to the *bc* plane. C—H⋯π(ring) inter­actions are indicated by dashed lines.

**Figure 3:**
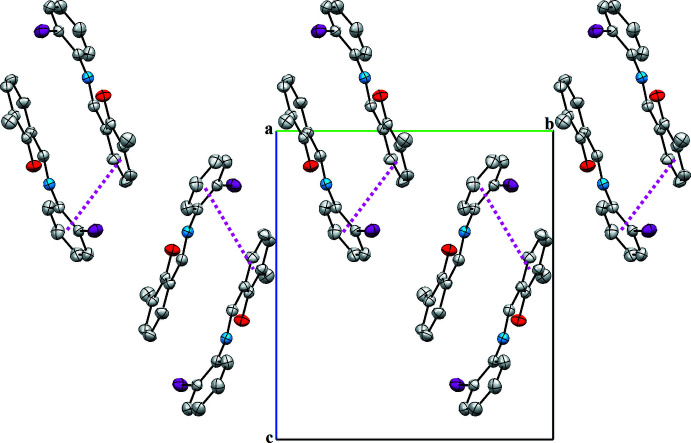
A view of the crystal packing of the title compound along the *a* axis. π(*Cg*1)⋯π(*Cg*2) inter­actions are indicated by dashed lines.

In the crystal structure, the mol­ecules are connected into sheets extending along the *a*-axis direction by C2—H2⋯*Cg*2^i^ inter­actions (Table 1 ▸; Fig. 2 ▸). Within the sheets, very weak π–π stacking inter­actions are observed with a centroid-to-centroid distance *Cg*1⋯*Cg*2^ii^ of 4.093 (2) Å (Fig. 3 ▸), where *Cg*1 and *Cg*2 are the centroids of the C1–C6 and C8–C13 rings, respectively.

## Hirshfeld surface analysis   

**Figure 4:**
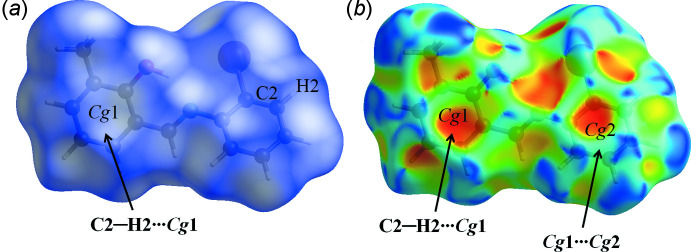
The Hirshfeld surfaces of the title compound mapped over (*a*) *d_norm_* and (*b*) shape-index.

**Figure 5:**
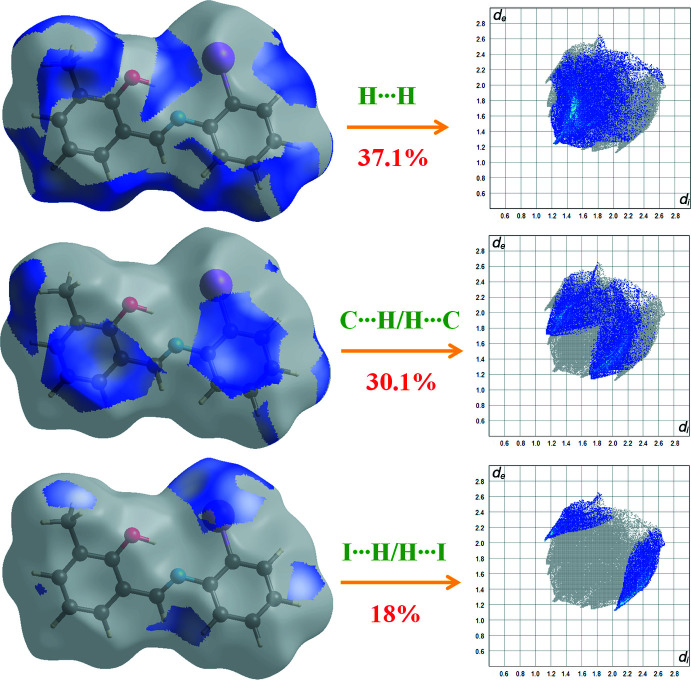
Two-dimensional fingerprint plots for the title compound, with a *d_norm_* view and the relative contribution of the atom pairs to the Hirshfeld surface.

A Hirshfeld surface analysis (Spackman & Jayatilaka, 2009 ▸) was carried out using *CrystalExplorer17.5* (Turner *et al.*, 2017 ▸). The Hirshfeld surfaces and the associated two-dimensional fingerprint plots were used to qu­antify the various inter­molecular inter­actions in the structure. The Hirshfeld surfaces (*d*_norm_ and shape-index) of the title compound are illustrated in Fig. 4 ▸. There are no prominent red spots on the surface, hence most of the inter­actions are weak non-covalent inter­actions. The diffuse white areas identified in Fig. 4 ▸ *a* and red areas on phenyl rings mapped with shape-index (Fig. 4 ▸ *b*) correspond to the H⋯π contacts resulting from hydrogen bond C—H⋯π(ring) (Table 1 ▸) and π–π stacking inter­actions. The major inter­molecular inter­actions in the crystal structure are H⋯H, H⋯C and H⋯I inter­actions, which make individual contributions of 37.1%, 30.1% and 18%, respectively. The fingerprint plots are shown in Fig. 5 ▸. There are also O⋯H (6.4%), N⋯H (3.6%) and C⋯C (23.3%) contacts. The Hirshfeld surface analysis confirms the importance of H-atom contacts in establishing the packing. The large number of H⋯H and C⋯H inter­actions suggest that van der Waals inter­actions play the major role in the crystal packing.

## Database survey   

A search of the Cambridge Structural Database (CSD, version 5.41, update of November 2019; Groom *et al.*, 2016 ▸) for the (*E*)-2-[(2-iodo­phenyl­imino)­meth­yl]phenol gave six hits: bis­[*N*-(2-iodo­phen­yl)-2-oxynaphthaldiminato-*N*,*O*]copper(II) (HABFIA; Unver, 2002 ▸), bis­(*m*-methano­lato)bis­(2-{[(5,7-di­iodo­quinolin-8-yl)imino]­meth­yl}phenolato)bis­(iso­thio­cyan­ato)­diiron(III) methanol solvate (HIDJOW; Sahadevan *et al.*, 2018 ▸), bis­(*m*-oxo)bis­(*m*-methano­lato)tetra­kis­(2-{[(5,7-di­iodo­quinolin-8-yl)imino]­meth­yl}phenolato)bis­(iso­thio­cyanato)­tetra­iron(III) di­chloro­methane solvate (HIDJUC; Sahadevan *et al.*, 2018 ▸), 2-{[(5,7-di­iodo­quinolin-8-yl)imino]­meth­yl}phenol (HIDKAJ; Sahadevan *et al.*, 2018 ▸), 2-iodo-salicylideneaniline (QQQANJ; Bernstein, 1967 ▸) and 2-[(2-iodo­phen­yl)imino­meth­yl]phenol (RAVTIR; Elmali & Elerman, 1997 ▸). In HABFIA, the C—O bond length is 1.293 (3) Å, compared to 1.339 (5) Å for this bond in RAVTIR. Similar values are observed in the crystal of the title compound. The C—N bond lengths are 1.306 (3) and 1.267 (5) Å in HABFIA and RAVTIR, respectively. The mol­ecules of HABFIA and RAVTIR have the same configuration as the title compound, while the other compounds listed above have different configurations.

## Synthesis and crystallization   

The title compound was prepared by refluxing mixed solutions of 2-hy­droxy-3-methyl­benzaldehyde (34.0 mg, 0.25 mmol) in ethanol (20 ml) and 2-iodo­aniline (54.7 mg, 0.25 mmol) in ethanol (20 ml). The reaction mixture was stirred for 4 h under reflux. Single crystals of the title compound for X-ray analysis were obtained by slow evaporation of an ethanol solution (yield 72%, m.p. 410–412 K).

## Refinement   

Crystal data, data collection and structure refinement details are summarized in Table 2 ▸. The C-bound H atoms were placed according to the difference-Fourier map and refined using a riding model: C—H = 0.93–0.96 Å with *U*_iso_(H) = 1.5*U*_eq_(C-meth­yl) and 1.2*U*_eq_(C) for other H atoms. Hydroxyl H atoms were placed according to a difference-Fourier map and were freely refined. The crystal studied was refined as a two-component inversion twin. This reflection file contains the non-overlapping reflections of the two twin components as well as the overlapping reflections. The BASF parameter for this two-component twin refined to −0.03242 (8).

## Supplementary Material

Crystal structure: contains datablock(s) I. DOI: 10.1107/S2056989020011974/zl2795sup1.cif

Structure factors: contains datablock(s) I. DOI: 10.1107/S2056989020011974/zl2795Isup2.hkl

Supporting information file. DOI: 10.1107/S2056989020011974/zl2795Isup3.cml

CCDC reference: 2026445

Additional supporting information:  crystallographic information; 3D view; checkCIF report

## Figures and Tables

**Table 1:** Hydrogen-bond geometry (Å, °) *Cg*2 is the centroid of the C8–C13 ring.

*D*—H⋯*A*	*D*—H	H⋯*A*	*D*⋯*A*	*D*—H⋯*A*
O1—H1⋯N1	0.93 (7)	1.82 (7)	2.634 (6)	144 (6)
C2—H2⋯*Cg*2^i^	0.93	2.97 (6)	3.7445 (4)	142 (4)

Symmetry code: (i) 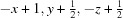.

**Table 2:** Experimental details

Crystal data	
Chemical formula	C_14_H_12_INO
*M*_r_	337.15
Crystal system, space group	Orthorhombic, *P*2_1_2_1_2_1_
Temperature (K)	296
*a*, *b*, *c* (Å)	8.1730 (4), 11.8143 (9), 13.1721 (8)
*V* (Å^3^)	1271.88 (14)
*Z*	4
Radiation type	Mo *K*α
μ (mm^−1^)	2.50
Crystal size (mm)	0.66 × 0.34 × 0.13

Data collection	
Diffractometer	Stoe *IPDS* 2
Absorption correction	Integration (*X-RED32*; Stoe & Cie, 2002 ▸)
*T*_min_, *T*_max_	0.365, 0.784
No. of measured, independent and observed [*I* > 2σ(*I*)] reflections	5163, 2482, 1949
*R*_int_	0.033
(sin θ/λ)_max_ (Å^−1^)	0.617

Refinement	
*R*[*F* ^2^ > 2σ(*F* ^2^)], *wR*(*F* ^2^), *S*	0.031, 0.063, 0.92
No. of reflections	2482
No. of parameters	160
H-atom treatment	H atoms treated by a mixture of independent and constrained refinement
Δρ_max_, Δρ_min_ (e Å^−3^)	0.46, −0.21

Computer programs: *X-AREA* and *X-RED* (Stoe & Cie, 2002 ▸), *SHELXT2017/1* (Sheldrick, 2015*a* ▸), *SHELXL2017/1* (Sheldrick, 2015*b* ▸), *PLATON* (Spek, 2020 ▸) and *WinGX* (Farrugia, 2012 ▸).

## supplementary crystallographic information

### Crystal data

**Table:**

C_14_H_12_INO	*D*_x_ = 1.761 Mg m^−^^3^
*M**_r_* = 337.15	Mo *K*α radiation, λ = 0.71073 Å
Orthorhombic, *P*2_1_2_1_2_1_	Cell parameters from 6431 reflections
*a* = 8.1730 (4) Å	θ = 2.5–32.6°
*b* = 11.8143 (9) Å	µ = 2.50 mm^−^^1^
*c* = 13.1721 (8) Å	*T* = 296 K
*V* = 1271.88 (14) Å^3^	Rod, orange
*Z* = 4	0.66 × 0.34 × 0.13 mm
*F*(000) = 656	

### Data collection

**Table:**

Stoe IPDS 2 diffractometer	2482 independent reflections
Radiation source: sealed X-ray tube, 12 x 0.4 mm long-fine focus	1949 reflections with *I* > 2σ(*I*)
Plane graphite monochromator	*R*_int_ = 0.033
Detector resolution: 6.67 pixels mm^-1^	θ_max_ = 26.0°, θ_min_ = 2.9°
rotation method scans	*h* = −8→10
Absorption correction: integration (X-RED32; Stoe & Cie, 2002)	*k* = −14→14
*T*_min_ = 0.365, *T*_max_ = 0.784	*l* = −12→16
5163 measured reflections	

### Refinement

**Table:**

Refinement on *F*^2^	Primary atom site location: structure-invariant direct methods
Least-squares matrix: full	Secondary atom site location: difference Fourier map
*R*[*F*^2^ > 2σ(*F*^2^)] = 0.031	Hydrogen site location: mixed
*wR*(*F*^2^) = 0.063	H atoms treated by a mixture of independent and constrained refinement
*S* = 0.92	*w* = 1/[σ^2^(*F*_o_^2^) + (0.0304*P*)^2^] where *P* = (*F*_o_^2^ + 2*F*_c_^2^)/3
2482 reflections	(Δ/σ)_max_ = 0.001
160 parameters	Δρ_max_ = 0.46 e Å^−^^3^
0 restraints	Δρ_min_ = −0.21 e Å^−^^3^

### Special details

**Table:**

Geometry. All esds (except the esd in the dihedral angle between two l.s. planes) are estimated using the full covariance matrix. The cell esds are taken into account individually in the estimation of esds in distances, angles and torsion angles; correlations between esds in cell parameters are only used when they are defined by crystal symmetry. An approximate (isotropic) treatment of cell esds is used for estimating esds involving l.s. planes.
Refinement. Refined as a two-component inversion twin.

### Fractional atomic coordinates and isotropic or equivalent isotropic displacement parameters (Å^2^)

**Table:**

	*x*	*y*	*z*	*U*_iso_*/*U*_eq_	
C1	0.5327 (7)	0.7863 (4)	0.8238 (6)	0.0512 (13)	
C2	0.6359 (10)	0.8192 (5)	0.9003 (6)	0.0664 (19)	
H2	0.598522	0.866780	0.951494	0.080*	
C3	0.7966 (11)	0.7810 (6)	0.9008 (6)	0.076 (2)	
H3	0.867715	0.803861	0.951899	0.092*	
C4	0.8492 (8)	0.7109 (5)	0.8273 (7)	0.0720 (18)	
H4	0.956517	0.684703	0.828468	0.086*	
C5	0.7452 (7)	0.6773 (5)	0.7497 (6)	0.0632 (17)	
H5	0.783507	0.629118	0.699235	0.076*	
C6	0.5849 (7)	0.7150 (5)	0.7469 (5)	0.0508 (14)	
C7	0.5196 (7)	0.6589 (5)	0.5815 (5)	0.0523 (13)	
H7	0.628176	0.672300	0.564200	0.063*	
C8	0.4100 (7)	0.6148 (4)	0.5043 (5)	0.0475 (14)	
C9	0.4719 (8)	0.5869 (5)	0.4092 (5)	0.0599 (17)	
H9	0.582202	0.598902	0.395435	0.072*	
C10	0.3738 (9)	0.5425 (5)	0.3361 (6)	0.0661 (17)	
H10	0.416758	0.523217	0.273023	0.079*	
C11	0.2093 (10)	0.5262 (5)	0.3564 (5)	0.0624 (17)	
H11	0.142297	0.496373	0.305986	0.075*	
C12	0.1420 (8)	0.5532 (5)	0.4495 (5)	0.0535 (15)	
C13	0.2432 (7)	0.5988 (4)	0.5232 (5)	0.0486 (15)	
C14	−0.0374 (8)	0.5358 (6)	0.4703 (7)	0.075 (2)	
H14A	−0.050482	0.493780	0.532175	0.113*	
H14B	−0.085832	0.494527	0.415201	0.113*	
H14C	−0.090242	0.607994	0.476842	0.113*	
I1	0.29233 (6)	0.84637 (4)	0.82323 (4)	0.07511 (17)	
O1	0.1754 (5)	0.6247 (4)	0.6139 (4)	0.0648 (12)	
N1	0.4728 (6)	0.6802 (3)	0.6727 (5)	0.0507 (11)	
H1	0.254 (8)	0.660 (5)	0.654 (6)	0.08 (2)*	

### Atomic displacement parameters (Å^2^)

**Table:**

	*U*^11^	*U*^22^	*U*^33^	*U*^12^	*U*^13^	*U*^23^
C1	0.050 (3)	0.050 (3)	0.053 (3)	−0.005 (2)	0.002 (4)	0.002 (3)
C2	0.074 (5)	0.058 (4)	0.067 (5)	−0.010 (3)	−0.003 (4)	−0.005 (3)
C3	0.073 (5)	0.075 (4)	0.081 (5)	−0.014 (4)	−0.025 (5)	−0.001 (4)
C4	0.058 (4)	0.068 (3)	0.090 (5)	−0.005 (3)	−0.015 (5)	−0.004 (5)
C5	0.050 (4)	0.057 (4)	0.083 (5)	0.002 (3)	−0.002 (3)	−0.007 (3)
C6	0.049 (4)	0.047 (3)	0.057 (4)	−0.002 (3)	−0.007 (3)	0.001 (3)
C7	0.049 (3)	0.050 (3)	0.058 (4)	0.002 (3)	0.008 (3)	0.001 (3)
C8	0.046 (3)	0.050 (3)	0.046 (3)	0.003 (2)	0.002 (3)	0.003 (2)
C9	0.054 (4)	0.070 (4)	0.056 (4)	−0.001 (3)	0.015 (3)	−0.003 (3)
C10	0.074 (5)	0.073 (4)	0.051 (4)	0.006 (4)	0.008 (4)	−0.012 (4)
C11	0.072 (4)	0.061 (3)	0.054 (4)	−0.001 (4)	−0.018 (4)	0.000 (3)
C12	0.049 (3)	0.057 (3)	0.055 (4)	−0.001 (3)	−0.006 (3)	0.005 (3)
C13	0.045 (4)	0.048 (3)	0.053 (3)	0.004 (2)	0.004 (3)	0.004 (3)
C14	0.053 (4)	0.089 (4)	0.084 (5)	−0.008 (4)	−0.009 (4)	0.006 (4)
I1	0.0656 (3)	0.0895 (3)	0.0703 (3)	0.0186 (2)	0.0053 (3)	−0.0094 (3)
O1	0.049 (3)	0.088 (3)	0.057 (3)	−0.002 (2)	0.009 (2)	−0.007 (2)
N1	0.047 (2)	0.050 (2)	0.055 (3)	−0.0006 (18)	−0.001 (3)	0.002 (3)

### Geometric parameters (Å, º)

**Table:**

C1—C2	1.370 (10)	C8—C9	1.390 (9)
C1—C6	1.385 (9)	C8—C13	1.399 (8)
C1—I1	2.089 (6)	C9—C10	1.359 (10)
C2—C3	1.389 (11)	C9—H9	0.9300
C2—H2	0.9300	C10—C11	1.384 (10)
C3—C4	1.344 (11)	C10—H10	0.9300
C3—H3	0.9300	C11—C12	1.382 (9)
C4—C5	1.387 (10)	C11—H11	0.9300
C4—H4	0.9300	C12—C13	1.384 (8)
C5—C6	1.384 (8)	C12—C14	1.505 (9)
C5—H5	0.9300	C13—O1	1.352 (7)
C6—N1	1.401 (8)	C14—H14A	0.9600
C7—N1	1.286 (8)	C14—H14B	0.9600
C7—C8	1.451 (9)	C14—H14C	0.9600
C7—H7	0.9300	O1—H1	0.93 (7)
			
C2—C1—C6	121.4 (6)	C10—C9—C8	121.0 (6)
C2—C1—I1	119.0 (5)	C10—C9—H9	119.5
C6—C1—I1	119.6 (5)	C8—C9—H9	119.5
C1—C2—C3	119.6 (7)	C9—C10—C11	119.3 (7)
C1—C2—H2	120.2	C9—C10—H10	120.3
C3—C2—H2	120.2	C11—C10—H10	120.3
C4—C3—C2	120.0 (7)	C12—C11—C10	121.8 (7)
C4—C3—H3	120.0	C12—C11—H11	119.1
C2—C3—H3	120.0	C10—C11—H11	119.1
C3—C4—C5	120.7 (7)	C11—C12—C13	118.3 (6)
C3—C4—H4	119.7	C11—C12—C14	121.2 (6)
C5—C4—H4	119.7	C13—C12—C14	120.5 (6)
C6—C5—C4	120.5 (6)	O1—C13—C12	117.6 (5)
C6—C5—H5	119.7	O1—C13—C8	121.7 (6)
C4—C5—H5	119.7	C12—C13—C8	120.7 (6)
C5—C6—C1	117.8 (6)	C12—C14—H14A	109.5
C5—C6—N1	122.9 (6)	C12—C14—H14B	109.5
C1—C6—N1	119.2 (5)	H14A—C14—H14B	109.5
N1—C7—C8	122.8 (6)	C12—C14—H14C	109.5
N1—C7—H7	118.6	H14A—C14—H14C	109.5
C8—C7—H7	118.6	H14B—C14—H14C	109.5
C9—C8—C13	118.9 (6)	C13—O1—H1	109 (4)
C9—C8—C7	119.4 (6)	C7—N1—C6	121.0 (5)
C13—C8—C7	121.7 (6)		
			
C6—C1—C2—C3	0.4 (10)	C8—C9—C10—C11	0.9 (10)
I1—C1—C2—C3	−179.2 (5)	C9—C10—C11—C12	−0.5 (10)
C1—C2—C3—C4	−0.9 (11)	C10—C11—C12—C13	0.8 (9)
C2—C3—C4—C5	0.9 (11)	C10—C11—C12—C14	179.6 (6)
C3—C4—C5—C6	−0.3 (11)	C11—C12—C13—O1	180.0 (5)
C4—C5—C6—C1	−0.3 (9)	C14—C12—C13—O1	1.2 (8)
C4—C5—C6—N1	−177.3 (6)	C11—C12—C13—C8	−1.4 (8)
C2—C1—C6—C5	0.2 (9)	C14—C12—C13—C8	179.8 (5)
I1—C1—C6—C5	179.8 (5)	C9—C8—C13—O1	−179.7 (5)
C2—C1—C6—N1	177.4 (5)	C7—C8—C13—O1	0.7 (8)
I1—C1—C6—N1	−3.1 (8)	C9—C8—C13—C12	1.7 (8)
N1—C7—C8—C9	−175.8 (6)	C7—C8—C13—C12	−177.9 (5)
N1—C7—C8—C13	3.8 (9)	C8—C7—N1—C6	175.4 (5)
C13—C8—C9—C10	−1.5 (9)	C5—C6—N1—C7	−33.7 (9)
C7—C8—C9—C10	178.2 (6)	C1—C6—N1—C7	149.2 (5)

### Hydrogen-bond geometry (Å, º)

*Cg*2 is the centroid of the C8–C13 ring.

**Table:**

*D*—H···*A*	*D*—H	H···*A*	*D*···*A*	*D*—H···*A*
O1—H1···N1	0.93 (7)	1.82 (7)	2.634 (6)	144 (6)
C2—H2···*Cg*2^i^	0.93	2.97 (6)	3.7445 (4)	142 (4)

Symmetry code: (i) −*x*+1, *y*+1/2, −*z*+1/2.
